# The Spatial Relationship of Malignant and Benign Breast Lesions with Respect to the Fat-Gland Interface on Magnetic Resonance Imaging

**DOI:** 10.1038/srep39085

**Published:** 2016-12-14

**Authors:** Won Hwa Kim, MuLan Li, Wonshik Han, Han Suk Ryu, Woo Kyung Moon

**Affiliations:** 1 Department of Radiology, Seoul National University College of Medicine and Seoul National University Hospital, Seoul, Korea; 2 Department of Radiology, Kyungpook National University Medical Center, Daegu, Korea; 3 International Radiology Imaging System, ShenZhen Mindray Bio-Medical Electronics Co., LTD., Shenzhen 518057, China; 4 Department of Surgery, Seoul National University College of Medicine, Seoul, Korea; 5 Department of Pathology, Seoul National University College of Medicine, Seoul, Korea

## Abstract

The fat-gland interface in the breast is noteworthy in that major vessels and lymphatic channels supplying the breast are located there; however, the relationship between breast lesion formation and the fat-gland interface is poorly understood. Here we evaluate the location of malignant and benign breast lesions with respect to the fat-gland interface in 881 women 50 years of age and younger, utilizing MR imaging. We find that most breast lesions are located in or near the interface in qualitative (89.7%) and quantitative (90.0%, 1 cm within the interface) analyses. This propensity for the fat-gland interface is not accounted for by breast anatomy, whereby 12.3% and 55.7% of breast volume is within 2 mm and 1 cm of the interface, respectively. Malignant lesions were located in or near the interface in significantly higher proportions than benign lesions in qualitative (94.3% vs. 67.3%, *P *< 0.001) and quantitative (49.7% vs. 34.5%, *P* < 0.001, 2 mm within the interface) analyses. This phenomenon may reflect a biological importance of the fat-gland interface in breast cancer development and progression.

In the breast, most pathology arises from the terminal ductolobular unit (TDLU) which is located in glandular tissue^1^,^2^,^3^. Glandular tissue abuts subcutaneous and retromammary fat, establishing the fat-gland interface, a region containing major blood vessels and lymphatic channels, as well as the anterior mammary fascia, which may act as a barrier to cancer invasion^2^,^4^.

A previous review of mammograms in women younger than 50 years of age found that most mammographically detected cancers (73%, 63 of 86) were located at the periphery of the parenchymal cone, which was defined by a 1-cm wide zone beneath the subcutaneous or retromammary fat^5^. These researchers concluded that geometric factors, rather than fat or biological considerations, might explain the peripheral location of breast cancers. They assumed that the breast and breast parenchyma were hemispheres and calculated the volume of breast parenchyma within the 1-cm zone of the parenchymal cone. In that study, however, the determination of lesion location was based on two mammography views, few lesions were included, and the study did not include benign lesions for comparison. Furthermore, the breast size used in the geometric explanation was estimated from the brassiere size.

Magnetic resonance (MR) imaging has an unprecedented ability to evaluate breast anatomy and focal lesions in three dimensions (3D)^6^. MR imaging enables evaluation of the 3D relationship between the surrounding fat or glandular tissue and breast lesions. Given the high resolution available with MRI and 3D capabilities, MR imaging has been widely used for screening in high risk women, for evaluation of extent of disease in patients with breast cancer receiving neoadjuvant chemotherapy, and for further evaluation of indeterminate breast lesions identified on mammogram and ultrasound^6^. However, the locations of malignant and benign lesions in the breast have rarely been investigated^7^,^8^. We hypothesize that malignant breast lesions, particularly invasive breast cancers, have a predilection for the fat-gland interface as compared to benign breast lesions^9^.

The purpose of this study was to investigate the location of malignant and benign breast lesions in relation to the fat-gland interface in women 50 years of age and younger on MR imaging.

## Materials and Methods

### Subjects

**Figure 1:**
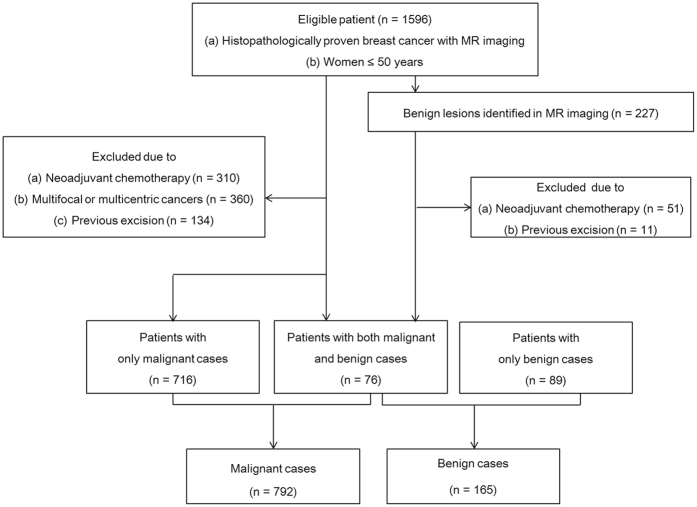
Flow chart of case selection for study.

This retrospective study received institutional review board (IRB) approval (Seoul National University Hospital, IRB No.1207-029-416), and the requirement for informed consent was waived. Between January 2009 and March 2013, 1596 consecutive women who were 50 years of age and younger underwent breast MR imaging for histopathologically proven breast cancer. We chose a study population of relatively younger patients (≤50 years) because these patients usually possess enough glandular tissue to investigate the spatial relationship between the breast lesions and the surrounding subcutaneous or retromammary fat and glandular tissue. We excluded 804 patients in which lesion location could not be determined due to: (a) prior neoadjuvant chemotherapy (n = 310); (b) multifocal or multicentric breast cancer (n = 360); and/or (c) prior excisional biopsy or breast surgery (n = 134). Suspicious lesions in the contralateral breast identified on MR imaging and confirmed benign on core needle biopsy or excisional biopsy during surgery were used as controls. As a result, a total of 881 women (mean, 43 yrs; age range, 21–50 yrs) with 792 malignant lesions and 165 benign lesions comprised our study group (Fig. 1). Malignant lesions for 299 of the 792 patients (37.8%) were initially detected on screening mammography, while the remaining were detected on diagnostic imaging performed for a palpable mass (n = 467, 59.0%) or nipple discharge (n = 26, 3.3%).

### MR Imaging Acquisition

MR imaging was performed with the patient in the prone position. All MR examinations were performed using a 1.5-T MR imager (Signa; GE Medical Systems, Milwaukee, WI) with a dedicated 8-channel bilateral breast coil (GE Medical Systems). Sagittal fat-suppressed, T2-weighted, fast-spin-echo MR imaging was performed using the following imaging parameters: repetition time msec/echo time msec, 5500–7150/85.2; matrix, 256 × 160; field of view, 200 × 200 mm; section thickness, 1.5 mm; and no gap. Dynamic contrast material–enhanced MR examinations included one pre-contrast and five post-contrast examinations with bilateral sagittal image acquisition using a fat-suppressed, T1-weighted, 3D fast spoiled gradient-echo sequence with parallel imaging (6.5/2.5; matrix, 256 × 160; flip angle, 10°; field of view, 200 × 200 mm; section thickness, 1.5 mm; and no gap). The acquisition time of each post-contrast series was 76 seconds. Five post-contrast image series were obtained at 91, 180, 360, 449, and 598 seconds after the initiation of contrast agent administration. Gadobenate dimeglumine (Multihance; Bracco Imaging, Milan, Italy) was injected into an antecubital vein using an automated injector (Spectris Solaris; Medrad Europe, Maastricht, the Netherlands) at 0.1 mmol/kg and at a rate of 2 mL/sec, followed by a 20-mL saline flush. Early subtraction (i.e., first post-contrast images minus pre-contrast images), axial reformatted images, and 3D maximum intensity projection (MIP) images were generated for all studies.

### MR Image Analysis

Two breast radiologists (W.H.K. and W.K.M., with 6 and 15 years of experience in breast MR imaging, respectively) performed a retrospective review of the location of malignant and benign lesions relative to the fat-gland interface in the breast in consensus. Information on lesion locations was obtained using clock-face positioning and size by MR imaging and provided to the radiologists to allow lesion-by-lesion analyses. The radiologists were blinded to the clinicopathological data (e.g., malignant and benign lesions). The entire series of MR images, including axial reformatted and MIP images, were comprehensively reviewed using a picture archiving and communication system (PACS) workstation. These two radiologists determined the amount of fibroglandular tissue (FGT; almost entirely fat, scattered FGT, heterogeneous FGT, or extreme FGT) according to the fifth edition of the Breast Imaging Reporting and Data System (BI-RADS) MR imaging lexicon^10^.

**Figure 2:**
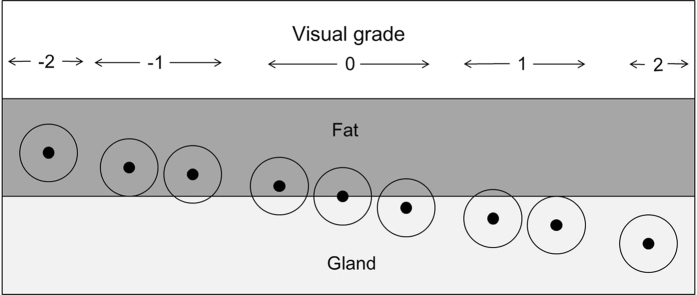
Schematic drawing of the classification of the lesions according to the spatial relationship with the fat-gland interface in the breast. The gray area indicates the fat tissues consisting of subcutaneous and retromammary fat, and the white area indicates the glandular tissue. Breast lesions were graded according the spatial relationship with the fat-gland interface in the breast as 2 = within the glandular tissue, 1 = near the fat-gland interface, glandular side, 0 = the fat-gland interface, −1 = near the fat-gland interface, fat side, −2 = within the fat tissue.

For quantitative analyses, the radiologists visually graded the breast lesions based on their spatial relationships with the fat-gland interface in the breast using the following five categories: 2 = within the gland; 1 = near the fat-gland interface, gland side; 0 = fat-gland interface; −1 = near the fat-gland interface, fat side; and −2 = within the fat (Fig. 2). A lesion within the gland was defined as a lesion with all surfaces surrounded by glandular tissue. A lesion near the fat-gland interface, gland side, was assigned when the distance between the center of the lesion and the fat-gland interface was the same or larger than the half-radius of the lesion. A lesion in the fat-gland interface was defined when the center of the lesion was on the fat-gland interface or the distance between the center of the lesion and the fat-gland interface was smaller than the half-radius of the lesion. A lesion near the fat-gland interface, fat side, was defined when the distance between the center of the lesion and fat-gland interface was the same or larger than the half-radius of the lesion. A lesion within the fat was assigned when the lesion surface was surrounded by fat tissue.

Quantitative analysis was performed to ensure the accurate classification of the spatial relationship of the breast lesions and to overcome subjectivity. One author (M.L., with 4 years of experience), who was not involved in the qualitative analysis, measured the shortest distance between the center of the lesion and the fat-gland interface on the sagittal or axial image. A positive value was assigned when the lesion center was in the gland tissue, and a negative value was assigned when the lesion center was in the fatty tissue. The measurement was performed using the image plane in which the longest diameter of the lesion was shown. The lesions in our study included mass (n = 835, 87.3%) and non-mass enhancement (n = 122, 12.7%). The location of the lesion, including mass and non-mass enhancement, was based on the center of the lesion from the point of the intersection of the major and minor axis of the lesion, which was usually perpendicular to the major axis.

After a minimum of 2 months since the first review, the reviewers re-reviewed 188 randomly selected lesions (159 malignant lesions and 29 benign lesions) to assess the intraobserver variability of the qualitative and quantitative analyses.

### Histopathological Review

A pathologist with 25 years of experience performed the histopathological diagnoses. The extent of the invasive portion and *in situ* cancer portion was also provided for invasive ductal carcinoma (IDC) with ductal carcinoma *in situ* (DCIS). Lesions with IDC were subdivided by the extent of associated DCIS, as follows: IDC without extensive DCIS (ratio of tumor size involving DCIS/invasive tumor size < 2), and IDC with extensive DCIS (ratio of tumor size involving DCIS/invasive tumor size ≥ 2). We classified each diagnosis of benign breast lesions from the pathological results into one of three categories: Fibroadenoma (n = 59), non-proliferative (n = 42), and proliferative (n = 64) benign lesions modified from the WHO classification and other studies^11^,^12^. Non-proliferative lesions (n = 42) included fibrocystic changes (n = 40) and non-sclerosing adenosis (n = 2). Proliferative lesions (n = 64) included usual or florid ductal hyperplasia (n = 35), papilloma (n = 13), atypical ducal hyperplasia (n = 10), atypical lobular hyperplasia (n = 1), and lobular carcinoma *in situ* (n = 5). If there were multiple diagnoses on a single biopsy, we assigned the lesion showing the dominant pathology into a category.

### Mathematical Analyses

**Figure 3:**
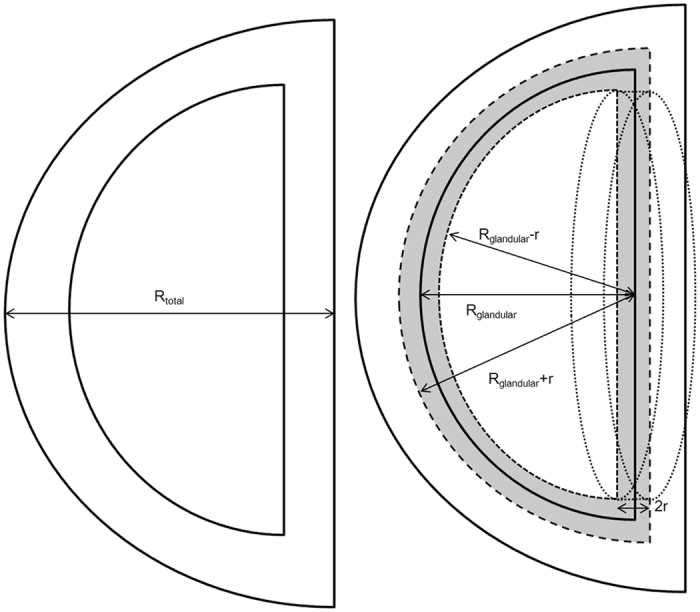
Schematic of a hemispheric breast. The light gray area indicates the interface zone surrounding the fat-gland interface. R_total_ = radius of the total breast, R_glandular_ = radius of the glandular tissue, r = size of the interface zone.

We calculated the percentage of the interface zone in the breast using a hemispheric model according to the estimated breast radius and size and then defined the sizes of interface zones according to a previous method, with modifications^5^. The radius of the hemisphere of the total breast and glandular portion was estimated in this hemispheric model from the mean value of the 3D breast diameter measured on sagittal and axial MR images (Fig. 3).

The volume of interface (V_I_) with a certain size of interface zone (r) around the radius of glandular tissue (R_glandular_) was calculated by subtracting the volume of the hemisphere with a radius of “R_glandular_ − r” from the volume of the hemisphere from the volume of the hemisphere with a radius of “R_glandular_ + r”, followed by the addition of the volume of a cylinder with a radius of “R_glandular_ − r” and a height of 2r. The formula of the interface volume (V_I_) was given as follows:


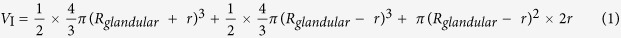


The total breast volume (V_T_) was calculated using the following formula:


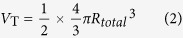


for the volume of a hemisphere with an estimated radius of R_total_. Therefore, the final percentage of V_I_ of the V_T_ was calculated using each interface size (2 mm, 5 mm, and 1 cm).

### Statistical Analyses

We recorded the assessment categories of the breast lesion location by radiologists and calculated the percentages of breast lesions within 2 mm, 5 mm, and 1 cm from the fat-gland interface. Student’s *t*-test and a chi-squared test were used to determine differences in the spatial relationships based on pathological features. One-way analysis of variance (ANOVA) and Bonferroni-Dunn post-hoc tests were performed for comparisons of more than two groups. Subgroup analysis was performed for screening-detected malignant lesions and small lesions (up to 1 cm on MR imaging). We tested the null hypothesis that the percentages of the lesions within 2 mm, 5 mm, and 1 cm observed in our study were the same as the percentage volume calculated from the geometry of the breast in a hemispheric model. Intraobserver variability was assessed using intraclass correlation coefficients (ICC). All statistical analyses were performed using SPSS version 12.0 software (SPSS, Chicago, IL) and GraphPad InStat (version 3.05, San Diego, CA). A *P* value less than.05 was considered to indicate statistical significance.

## Results

The mean diameter of invasive breast cancers on MR imaging was 2.0 cm (standard deviation [SD], 1.2 cm), and pure DCIS on histology was 3.5 cm (SD, 2.2 cm). The mean size of benign lesions recorded from MR imaging was 1.3 cm (SD, 0.8 cm). The amount of FGT was classified as entirely fatty in 0.8% (n = 8), scattered in 6.6% (n = 63), heterogeneous in 59.5% (n = 569), and extreme in 33.1% (n = 317) of subjects.

**Figure 4:**
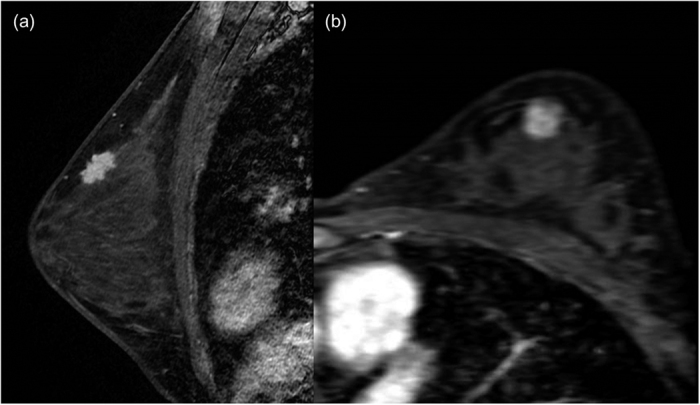
An infiltrating ductal carcinoma with focal ductal carcinoma *in situ* in the left breast of a 38-year-old woman. (**a**) Sagittal contrast-enhanced, non-subtracted, T1-weighted MR image with fat suppression shows a 2 cm spiculated mass. Note the tumor location in the fat-gland interface. (**b**) Subtracted axial reformatted image.

**Figure 5:**
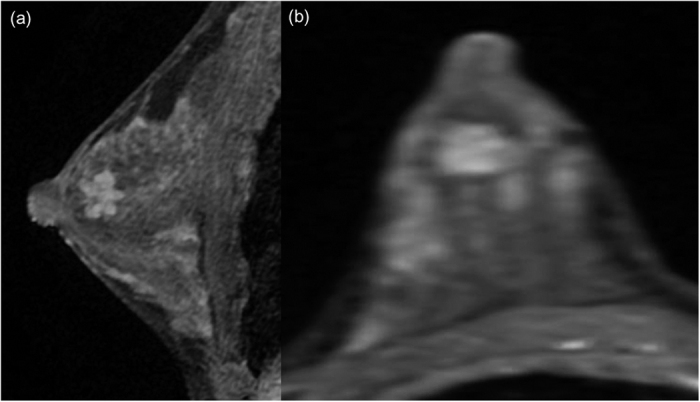
A fibroadenoma in the right breast of a 26-year-old woman. (**a**) Sagittal contrast-enhanced non-subtracted T1-weighted MR image with fat suppression shows a 1.5 cm circumscribed mass. Note the mass located within the glandular tissue. (**b**) Subtracted axial reformatted image.

Most lesions (89.7%, 858/957) were located in or near the fat-gland interface, with malignant lesions having a greater propensity to be in or near the interface compared to benign lesions (94.3% [747/792] vs. 67.3% [111/165], *P* < 0.001; Fig. 4). Benign lesions were more often located within glandular tissue compared to malignant lesions (29.7% [49/165] vs. 5.1% [40/792], *P* < 0.001; Fig. 5). There was a higher proportion of pure DCIS near the interface, gland side, compared to IDC, invasive lobular carcinoma or IDC with extensive DCIS (70.0% [49/70] vs. 28.8% [138/479], 36.2% [17/47], and 48.0% [94/196], *P* < 0.001, *P* = 0.002, and *P* < 0.001, respectively). There was a higher proportion of lesions at or near the interface on the fat side, and a lower proportion of lesions near the interface, gland side, for IDC and invasive lobular carcinoma, as compared to IDC with extensive DCIS or pure DCIS. For benign lesions, there was no significant difference between proliferative and non-proliferative lesions (*P* = 0.188). Table 1 describes the detailed qualitative analyses according to the pathological types of malignant and benign lesions.

In the quantitative analysis, 47.1% (451/957, 95% confidence interval [CI]: 43.9% to 50.3%), 68.4% (655/957, 95% CI: 65.3% to 71.3%), and 90.0% (861/957, 95% CI: 87.9% to 91.8%) of the breast lesions were located within 2 mm, 5 mm, and 1 cm, respectively, from the fat-gland interface (Table 2). Compared to benign lesions, malignant lesions tended to be present in higher proportions within 2 mm (49.7% [394/792] vs. 34.5% [57/165]), 5 mm (70.3% [557/792] vs. 59.4% [98/165]), and 1 cm (91.2% [722/792] vs. 84.2% [139/165]) from the fat-gland interface (*P* < 0.001, *P* = 0.007, and *P* = 0.010 for 2 mm, 5 mm, and 1 cm, respectively). A higher proportion of IDC or invasive lobular carcinoma lesions tended to be within 2 mm compared with IDC with extensive DCIS or pure DCIS lesions. For benign lesions, there were no significant differences between proliferative and non-proliferative lesions in the proportion of lesions within 2 mm, 5 mm, and 1 cm (*P* = 0.532, *P* = 0.547, and *P* = 0.281, respectively).

The estimated radii of the glandular tissue and total breast were 4.5 cm (SD, 2.2 cm) and 6.6 cm (SD, 1.9 cm), respectively. The percentages of breast volumes within 2 mm, 5 mm, and 1 cm from the fat-gland interface were calculated from the total breast volume, which was based on this estimated radius, as 12.3%, 29.6%, and 55.7%, respectively. The results of subgroup analyses on screening-detected malignant lesions (n = 299) and small lesions (up to size 1 cm on MR imaging) (n = 171) were similar to those of the original analysis (Tables S1–S4).

For the null hypothesis, statistical tests revealed that both malignant and benign lesions were located 2 mm, 5 mm, and 1 cm from the fat-gland interface, more often than could be accounted for by the geometric configuration of breast tissue (all *P*s < 0.001)

The ICC value between repeated measurements in the quantitative and qualitative analyses was 0.697 (95% CI 0.616, 0.764) and 0.733 (95% CI 0.659, 0.793), respectively, which indicated substantial intraobserver agreement.

## Discussion

This study evaluated the location of malignant and benign breast lesions in respect to the fat-gland interface in women 50 years of age or younger, using 3D MR imaging. Our results showed that most of the lesions were located in or near the fat-gland interface in qualitative (89.7%) and quantitative (90.0%, 1 cm within the interface) analyses; notably, a greater proportion of malignant lesions were located in this area. This finding is consistent with a previous study by Stacy-Clear *et al*., who reported that most cancers detected by mammography are peripherally located^5^. Stacy-Clear *et al*. concluded that lesion location was secondary to breast geometry, with the peripheral zone simply accounting for a larger breast volume. With the availability of MRI, we are now able to appreciate 3D breast anatomy, allowing a more accurate calculation of the fat-glandular interface and breast volume, as opposed to estimations from brassiere size and mammogram location (methods previously employed). Our estimation and statistical testing did not satisfy the null hypothesis of chance resulting from a geometric configuration. This result suggests that the fat-gland interface location of breast cancer is not due to chance, but instead reflects a biological phenomenon.

In our study, malignant lesions exhibited a stronger tendency to be located in or near the fat-gland interface. Notably, more invasive cancerous lesions and fewer DCIS lesions tended to be located in the fat-gland interface. These findings might support the recent understanding that fat plays an important role in breast cancer development and progression^9^,^13^,^14^,^15^. Hormones, growth factors, and cytokines known as adipokines, which are secreted by fat tissue, stimulate the growth and proliferation of breast tumor cells^16^,^17^. Tumor-surrounding adipocytes exhibit phenotypic changes and promote tumor cell invasion and metastasis^18^. These physiologic influences affect where and how disease arises and spreads^19^,^20^. Malignant lesions arise at the junction of extralobular terminal duct with the lobule, and the axis of its growth is perpendicular to the long axis of the lobule. In contrast, fibroadenoma arises within the lobule, and it is forced into a growth axis that is parallel to the long axis of the lobule^6^. This observation has been reported using MR imaging and ultrasound and has been widely used in the differentiation of solid breast masses^2^,^19^,^21^.

The predisposition of breast lesions to localize to the fat-gland interface, as demonstrated in this study, reinforces the need for careful evaluation of this region when interpreting breast imaging studies using MR imaging, mammography, and ultrasound^22^,^23^,^24^,^25^. In one study, a retrospective evaluation of the prior MR imaging studies in patients with breast cancer detected a potential observer error in 47% of cases^22^. An interface-visualized anterior mammary fascia is recognizable in breast ultrasound and mammography, and the penetration of this interface might imply that the lesion is invasive^26^,^27^. Our results support the development of techniques to better image the fat-gland interface of the breast^5^, which might result in earlier and improved detection of invasive breast cancer.

We acknowledge several limitations of our study. First, this was a retrospective study to investigate the location of breast lesions and their spatial relationships with the fat-gland interface, which was assessed qualitatively and quantitatively using 3D MR images. These measurements are potentially prone to inter- and intraobserver variability. The ICC values between repeated measurements of the quantitative and qualitative analyses, however, were 0.697 and 0.733, respectively, which indicated substantial intraobserver agreement. Second, typical benign lesions that did not require tissue confirmation were not included. A possible bias exists in the methods, whereby larger lesions were more likely to be considered to be located within or near the interface. However, the results of subgroup analyses of screening-detected malignant lesions and small lesions (up to size 1 cm on MR imaging) were similar to those of the original analysis. We assume that the TDLU near the fat-gland interface might have stem cell characteristics that are targeted by carcinogenic insults, leading to transformation and cancer^28^,^29^. The interaction between the fat and gland tissue near the fat-gland interface should be important in both normal development and neoplasia^30^,^31^. Further study is needed to investigate this hypothesis.

In conclusion, most breast lesions in women 50 years of age and younger were located in or near the fat-gland interface, and a higher proportion of malignant compared to benign lesions was observed in this region. This phenomenon is not fully explained by the geometric configuration of the breast, and may reflect biological importance of the fat-gland interface in breast cancer development and progression.

## Additional Information

**How to cite this article**: Kim, W. H. *et al*. The Spatial Relationship of Malignant and Benign Breast Lesions with Respect to the Fat-Gland Interface on Magnetic Resonance Imaging. *Sci. Rep.* **6**, 39085; doi: 10.1038/srep39085 (2016).

**Publisher's note:** Springer Nature remains neutral with regard to jurisdictional claims in published maps and institutional affiliations.

## Figures and Tables

**Table 1:** Qualitative Analysis of Lesion Location according to Spatial Relationship with the Fat-Gland Interface.

Pathological Type	N	Within Gland	Near Interface, Gland Side	Interface	Near Interface, Fat Side	Within Fat
Malignant lesions	**792**	**40 (5.1%)**	**298 (37.6%)**	**429 (54.2%)**	**20 (2.5%)**	**5 (0.6%)**
IDC without extensive DCIS*	479	32 (6.7%)	138 (28.8%)	285 (59.5%)	19 (4.0%)	5 (1.0%)
IDC with extensive DCIS*	196	5 (2.6%)	94 (48.0%)	96 (49.0%)	1 (0.5%)	0
Pure DCIS	70	1 (1.4%)	49 (70.0%)	20 (28.6%)	0	0
Invasive lobular carcinoma	47	2 (4.3%)	17 (36.2%)	28 (59.6%)	0	0
Benign lesions	**165**	**49 (29.7%)**	**56 (33.9%)**	**49 (29.7%)**	**6 (3.6%)**	**5 (3.0%)**
Fibroadenoma	59	19 (32.2%)	19 (32.2%)	16 (27.1%)	2 (3.4%)	3 (5.1%)
Non-proliferative**	42	11 (26.2%)	12 (28.6%)	14 (33.3%)	3 (7.1%)	2 (4.8%)
Proliferative**	64	19 (29.7%)	25 (39.1%)	19 (29.7%)	1 (1.6%)	0
*P* value		<0.001	0.425	<0.001	0.423	0.017

Note.—Data are numbers of patients and numbers in parentheses are percentages. IDC = invasive ductal carcinoma, DCIS = ductal carcinoma *in situ*.

*IDC without extensive DCIS was defined as the ratio of tumor size involving DCIS/invasive tumor size <2, and IDC with extensive DCIS was defined as the ratio of tumor size involving DCIS/invasive tumor size ≥ 2.

**Non-proliferative benign lesions included fibroadenomas, fibrocystic changes, and non-sclerosing adenosis. Proliferative without atypia included usual ductal hyperplasia, sclerosing adenosis, and papillomas. Proliferative with atypia included atypical ductal hyperplasia, atypical lobular hyperplasia, and lobular carcinoma *in situ*.

**Table 2:** Quantitative Analysis of Lesion Location according to Spatial Relationship with the Fat-Gland Interface

Pathological Type	N	Within 2 mm	Within 5 mm	Within 1 cm
Malignant lesions	**792**	**394 (49.7%)**	**557 (70.3%)**	**722 (91.2%)**
IDC without extensive DCIS*	479	256 (53.4%)	350 (73.1%)	440 (91.9%)
IDC with extensive DCIS*	196	89 (45.4%)	123 (62.8%)	172 (87.8%)
Pure DCIS	70	22 (31.4%)	48 (68.6%)	66 (94.3%)
Invasive lobular carcinoma	47	27 (57.4%)	36 (76.6%)	44 (93.6%)
Benign lesions	**165**	**57 (34.5%)**	**98 (59.4%)**	**139 (84.2%)**
Fibroadenoma	59	21 (35.6%)	34 (57.6%)	50 (84.7%)
Nonproliferative**	42	16 (38.1%)	27 (64.3%)	33 (78.6%)
Proliferative**	64	20 (31.2%)	37 (57.8%)	56 (87.5%)
*P* value		<0.001	0.007	0.010

Note.—Data are numbers of patients and numbers in parentheses are percentages. IDC = invasive ductal carcinoma, DCIS = ductal carcinoma *in situ*.

^*^IDC without extensive DCIS was defined as the ratio of tumor size involving DCIS/invasive tumor size <2, and IDC with extensive DCIS was defined as the ratio of tumor size involving DCIS/invasive tumor size ≥ 2.

^**^Non-proliferative benign lesions included fibroadenomas, fibrocystic changes, and non-sclerosing adenosis. Proliferative without atypia included usual ductal hyperplasia, sclerosing adenosis, and papillomas. Proliferative with atypia included atypical ductal hyperplasia, atypical lobular hyperplasia, and lobular carcinoma *in situ*.
